# Distinguishable Colorimetric Biosensor for Diagnosis of Prostate Cancer Bone Metastases

**DOI:** 10.1002/advs.202303159

**Published:** 2023-10-15

**Authors:** Ming Li, Caiping Ding, Dong Zhang, Weiwei Chen, Zejun Yan, Zikang Chen, Zhiyong Guo, Longhua Guo, Youju Huang

**Affiliations:** ^1^ Department of Urology & Nephrology The First Affiliated Hospital of Ningbo University 59 Liuting Street Ningbo Zhejiang 315010 China; ^2^ College of Material Chemistry and Chemical Engineering Key Laboratory of Organosilicon Chemistry and Material Technology Ministry of Education Key Laboratory of Organosilicon Material Technology of Zhejiang Province Department Hangzhou Normal University Hangzhou Zhejiang 311121 China; ^3^ State Key Laboratory for Managing Biotic and Chemical Threats to the Quality and Safety of Agro‐products State Key Laboratory Base of Novel Functional Materials and Preparation Science School of Materials Science and Chemical Engineering Ningbo University Ningbo Zhejiang 315211 China; ^4^ College of Biological Chemical Sciences and Engineering Jiaxing University Jiaxing Zhejiang 314001 China

**Keywords:** alkaline phosphatase, color‐recombining enhancement, gold nanobipyramids, prostate cancer bone metastasis, rhodamine derivatives

## Abstract

Castration‐resistant prostate cancer (PCa) causes severe bone metastasis (BM), which significantly increases mortality in men with PCa. Imaging tests and radiometric scanning require long analysis times, expensive equipment, specialized personnel, and a slow turnaround. New visualization technologies are expected to solve the above problems. Nonetheless, existing visualization techniques barely meet the urgency for precise diagnosis because the human eyes cannot recognize and capture even slight variations in visual information. By using dye differentiated superposition enhancement colorimetric biosensors, an effective method to diagnose prostate cancer bone metastases (PCa‐BM) with excellent accuracy for naked‐eye quantitative detection of alkaline phosphatase (ALP) is developed. The biomarker ALP specific hydrolytic product ascorbic acid can be detected by rhodamine derivatives (Rd) as gold nanobipyramids (Au NBPs) are deposited and grown. Color‐recombining enhancement effects between Rd and Au NBPs significantly improved abundance. The 150 U L^−1^ threshold between normal and abnormal can be identified by color. And with color enhancement effect and double signal response, the ALP index is visually measured to diagnose PCa‐BM and provide handy treatment recommendations. Additionally, the proposed colorimetric sensing strategy can be used to diagnose other diseases.

## Graphical abstract

An effective method to diagnose PCa‐BM with excellent accuracy for naked‐eye quantitative ALP detection is developed. The biomarker ALP specific hydrolytic product ascorbic acid can be detected by Rd as Au NBPs are deposited and grown. With color enhancement effect and double signal response, the ALP index is visually measured to diagnose PCa‐BM and provide handy treatment recommendations.

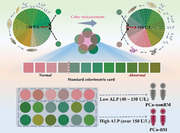

## 1 Introduction

About 1.3 million prostate cancer (PCa) cases are diagnosed each year worldwide. There will be 10 million PCa cases in 2021, and 0.7 million metastatic cases. Cancer‐related deaths from metastasis PCa are expected to double by 2040.^[^ ^1^ ^]^ Bone metastasis (BM) is an incurable PCa form with attendant high morbidity and mortality. BM involvement of the vertebral bodies can lead to pathologic fracture and spinal cord compression, resulting in significant neurologic and functional disabilities.^[^ ^2^ ^]^ PCa patients experience BM in 65% to 75% of cases.^[^ ^3^ ^]^ The progression of PCa to castration resistant PCa and BM will be further accelerated if patients do not receive timely diagnosis and treatment. It is unfortunate that BM causes metabolic disorders and serious complications, putting patients at risk of death. It is imperative to take effective measures to alleviate such a serious situation.^[^ ^4^ ^]^ The risk of disease deterioration can be reduced by timely BM diagnosis and controlling the severity of the disease. Identifying BM is also critical for PCa treatment effectiveness.

Imaging methods are commonly used to diagnose BM, including plain X‐rays, computed tomography (CT) scans, magnetic resonance imaging (MRI) and radionuclide bone scans (ECT).^[^ ^5^ ^]^ Radiation and allergies are risks associated with these methods. Moreover, these imaging assays require expensive precision instruments, and the detection process is time‐consuming and labor‐intensive, so patients' disease status cannot be monitored timely and effectively.^[^ ^6^ ^]^ It is therefore essential to develop a biosensor that is quick, labor‐saving, economical, easy‐to‐operate and easy‐to‐carry. Searching for various markers of metabolic BM has become a key research direction to solve these problems, since noninvasive diagnosis methods combined with biomarkers offer benefits such as low cost and short analysis times.^[^ ^7^ ^]^ Previous research reports showed that alkaline phosphatase (ALP) was a widely used biomarker for clinical BM monitoring with excellent specificity (98%) and sensitivity (60%).^[^ ^8^ ^]^ Elevated ALP levels are positively related to bone cancer growth, which is increased in osteoblastic osteopathies due to PCa cells multiplication.^[^ ^9^ ^]^ The occurrence of BM may increase serum ALP levels,^[^ ^10^ ^]^ which can be used to determine PCa‐BM expression levels compared to normal levels (40–150 U L^−1^).^[^ ^11^ ^]^

ALP activity assays include fluorometric,^[^ ^12^ ^]^ Raman scattering,^[^ ^13^ ^]^ capillary electrophoresis,^[^ ^14^ ^]^ electrochemical,^[^ ^15^ ^]^ chemiluminescence,^[^ ^16^ ^]^ and colorimetric.^[^ ^17^ ^]^ Research interest in colorimetric analysis has risen due to its ease of use, rapid analysis and low cost.^[^ ^18^ ^]^ In the development of advanced colorimetric sensing platforms,^[^ ^17d,e^ ^]^ plasmonic nanoparticles of various sizes and shapes have been used for visible color changes. Their plasmon resonance properties make them remarkable for detecting different analytes.^[^ ^19^ ^]^ ALP has been assessed with colorimetric methods using plasmonic nanoparticles. For example, the colorimetry based on gold nanospheres aggregation allowed semi‐quantitative analysis of ALP (1–50 U L^−1^) by separating red from purple;^[^ ^20^ ^]^ ALP concentration (5–100 U L^−1^) was determined by detecting multicolor changes with a silver nanoshell deposition on the surface of Au NRs.^[^ ^21^ ^]^ Due to the low intuitive level of color recognition at ≈150 U L^−1^, the ALP detection technology based on the above method cannot achieve critical color differentiation in PCa‐BM of ALP concentration. It is therefore worthwhile to further develop more facile and highly effective visual information processing techniques that can be used to detect abnormal ALP concentrations by the naked eyes for early diagnosis of PCa‐BM.

Medical treatment demands simpler and faster diagnostic methods. Vision plays a significant role in acquiring information and perceiving reality for humans. Color perception technology, including visualization technology, received recognition and attention. In spite of this, because human eyes are not capable of capturing and recognizing slight variations in visual information (like hue, brightness, saturation, shape, texture, size, and spatial location), existing visualization techniques are barely able to meet the urgent need for precise diagnosis because of their poor sensitivity and accuracy. Human eyes are remarkable for their excellent color discrimination capabilities, which makes color detection technology even more effective. To significantly improve naked‐eye quantitative detection sensitivity and accuracy, we proposed a color overlay method to produce significantly enriched stimuli‐responsive visual information. In order to maximize naked‐eye recognition, the analyte‐induced homogeneous hue change was converted into easily perceptible superimposed tones.^[^ ^22^ ^]^

Fortunately, previous reports had shown that important color parameters in color images such as red‐green‐blue (RGB) three‐channel intensity and CIELAB coordinates L*, a* and b* can be obtained by digital products for colorimetric analysis, and these parameters provide guidance for color overlay.^[^ ^23^ ^]^ Color distinguishable superposition may thus be used to enhance color signal discrimination for improving analytical performance.^[^ ^24^ ^]^ Zhang et al. proposed a dual‐parameter multiplexing strategy based on spatial separation and color co‐location.^[^ ^25^ ^]^ In this method, another parameter was added for multiplexing depending on the morphology and composition control of the three primary colors, thus realizing multi‐target analysis. Wang et al. proposed a concept of complex color light mixing, which generates infinite color combinations and capacities through chaotic light mixing and interaction behavior.^[^ ^26^ ^]^ Zhou et al. proposed a color palette based on binary photon pigments. It not only solved the dilemma of color mixing, but also enabled the tunability and multiplicity of colors, giving the world a new color palette to choose from.^[^ ^27^ ^]^ Color mixing was confirmed in the above studies, which makes us fully believe that color overlay improves the analytical performance of visual sensors.

**Figure 1:**
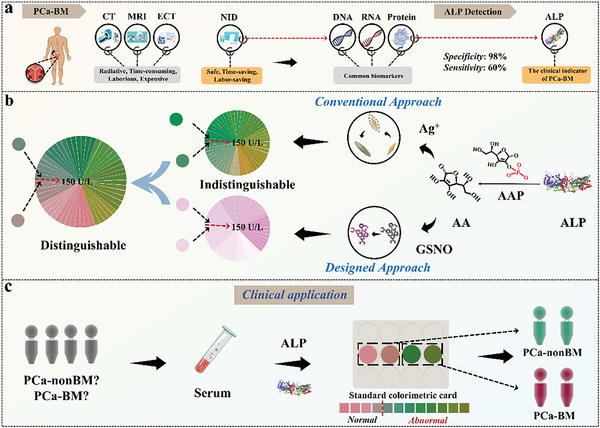
An illustration of a distinguishable colorimetric analysis of ALP concentration. a) ALP as a biomarker will replace PCa‐BM diagnosis. b) Mechanism and superiority of distinguishable colorimetry (DC). c) Monitoring and screening of PCa‐BM patients visually.

In this study, a colorimetric visualization method was designed for dye distinguishable superposition enhancement with a two‐signal response. The technique relied on dye mixing to distinguish critical concentrations clearly through color superposition, which can lead to a greater variety of, more controlled, and distinguishable visual information with a synergistic effect. **Figure** **1** shows ALP was first selected as the diagnostic marker for PCa‐BM through investigation and clinical analysis. Colorimetric visualization responses were induced based on ALP‐specific responses. Simultaneous steps, including ascorbic acid (AA) induced visualization of silver layer deposition on Au NBPs and Rd color development. However, the critical ALP value of 150 U L^−1^ cannot be intuitively identified by any color reaction to Au NBPs or Rd. Intriguingly, using color overlay principles and combining two colorimetric reactions, the naked eye's ability to distinguish between colors above and below the 150 U L^−1^ threshold was improved. An RGB‐based patterning code was presented for highly reliable color extraction and high‐density information diagnosis. Dye distinguishable superposition enhancement colorimetric visualization methods were successfully used to detect abnormally elevated ALP. It also enabled rapid diagnosis and screening of patients. This has significant development significance for PCa‐BM daily monitoring to remind patients of disease progress as soon as possible.

## 2 Results and Discussion

### 2.1 Design and Synthesis of Distinguishable Colorimetric Sensor Probe

**Figure 2:**
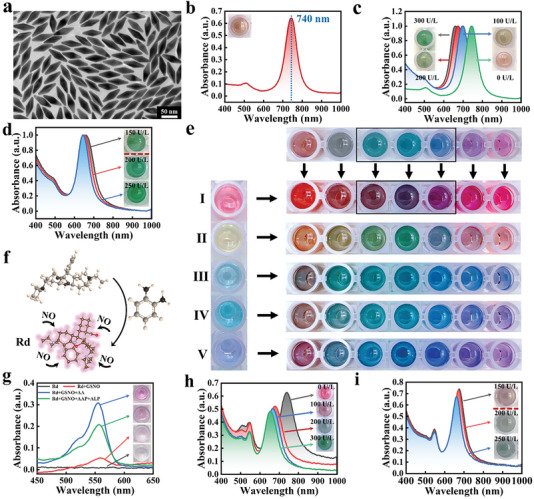
a) TEM and b) UV–vis absorption spectra of Au NBPs. c) UV–vis absorption spectra of Ag deposition and growth on Au NBPs in the presence of ALP at different concentrations (0, 100, 200, 300 U L^−1^) and color photographs of ANBPs (insets). d) UV–vis absorption spectra of conventional colorimetry and color differentiation at critical concentrations and color photographs of ANBPs (insets). e) Using five dyes, including Bengal rose red (I), methyl orange (II), methylene blue (III), fast green (IV), and ethyl violet (V), a nano‐gold solution was color modulated with different colors. f) Schematic diagram of Rd synthesis. g) UV–vis absorption spectra of before and after Rd recognition response, along with color photographs of Rd. h) UVvis absorption spectra of distinguishable colorimetric reactions in the presence of ALP at different concentrations (0, 100, 200, and 300 U L^−1^) and color photographs of mixed reaction solutions (insets). i) UV–vis absorption spectra of DC and color differentiation at critical concentrations and color photographs of mixed reaction solutions (insets).

To begin with, Au NBPs enabled a traditional silver deposition method on precious metal surfaces. As shown in **Figure** **2**a, purified homogeneous Au NBPs particles were used as one of the detection units (Figure 2b), with UV–vis absorption peaks at 740 nm. When ALP hydrolyzed L‐ascorbate‐2 phosphate magnesium salt hydrate (AAP) to produce AA, resulting in a gradual shift in the longitudinal local surface plasmon resonance (LSPR) peak to blue with changes in four colors (reddish brown, brown, dark green and bottle green) for Ag deposition and growth (Figure 2c). According to TEM characterization (Figure S2a–d, Supporting Information) and elemental analysis (Figure S2e–l, Supporting Information), the silver crust on the surface of Au NBPs gradually thicken and becomes rod‐like. However, it can be clearly seen from Figure 2d that when detecting the concentration range of 150–250 U L^−1^ by this method, the slight difference in green color cannot be effectively distinguished by the eyes, which limits its applicability to PCa‐BM early detection.

As we all know, nature's rich colors are produced by mixing and matching RGB's three primary colors. Color enhancement, such as the increase in color types, is more conducive to human eye discrimination. Inspired by this, our approach improved visual resolution by implementing a compound colorimetric visualization method. Hence, the color superposition enhancement conjecture was confirmed using five dyes, including Bengal rose red, methyl orange, methylene blue, fast green, and ethyl violet. These dyes were used to change the nano‐gold solution's color. In Figure 2e, compared with the original nano‐gold solution, the types and vividness of colors have changed significantly after dye addition, especially with red and yellow dyes. Furthermore, it was found that red dyes produced three distinct mixed colors after adding blue solution (Figure 2e‐I), indicating that the red dye was very suitable for enhancing visual resolution. The distinguishable colorimetric strategy will enhance visual recognition while improving detection applicability.

Based on this, a NO recognition unit which outputs a purplish red signal was synthesized according to Figure 2f. This unit was used for detection and color blending. The molecular structure of the synthesized product (Figure S1, Supporting Information) was determined using ^1^H‐NMR. As an initiation strategy for color mixing, the Rd recognition response was initiated when GSNO reacted with AA to produce NO. This ultimately caused the color to change from colorless to purplish red, with significant absorption changes at 550 nm (Figure 2g). In Figure 2h, it can be seen that the color was brighter after the distinct reaction of two paths, indicating a higher resolution than conventional colorimetry. In addition, with the addition of dye molecules, the color at 150 U L^−1^ changes from an indistinguishable green to a distinct distinguishable reddish brown and dark green. This points out that DC greatly enhances color differentiation at 150 U L^−1^ (Figure 2i). Results show that the color superposition enhancement effect can be achieved by simultaneously introducing two colors with marker ALP to achieve intuitive differentiation at 150 U L^−1^.

### 2.2 Optimization of Key Parameters of Distinguishable Colorimetric Sensor

**Figure 3:**
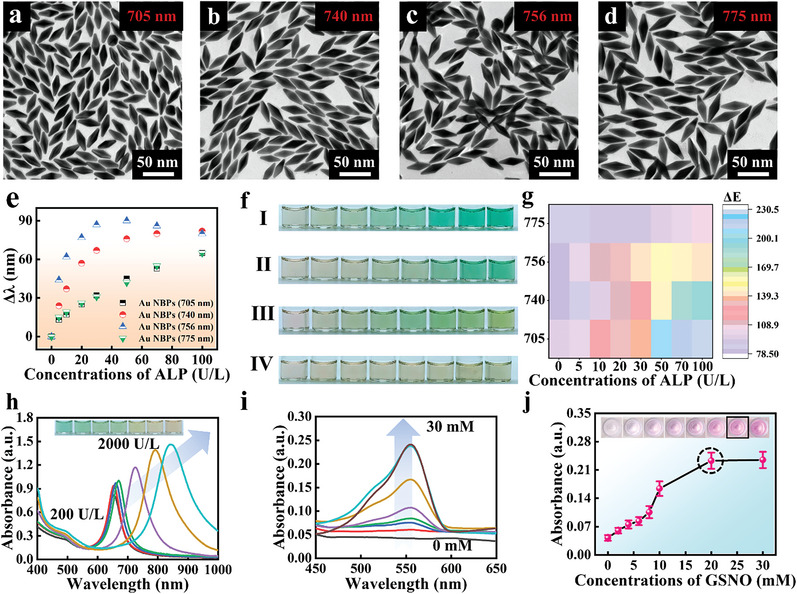
a–d) TEM images of Au NBPs at four wavelengths (705, 740, 756, and 775 nm). In the presence of ALP, e) LSPR peak displacement and f) color changes (square I: 705 nm, square II: 740 nm, square III: 756 nm, square IV: 775 nm) after silver deposition and growth on Au NBPs. g) Heat map of color difference calculated according to color changes in silver deposition and growth on Au NBPs. h) Displacement of UV–vis absorption spectra formed by excessive silver deposition and growth on Au NBPs (inset: color photographs). i) UV–vis absorption spectra and j) scatter diagram of GSNO concentration optimization (inset: color photographs).

Distinguishable colorimetric strategy parameters were optimized for optimal detection performance. It is crucial to select Au NBPs in colorimetric reactions due to color differences.^[^ ^28^ ^]^ The reason is that Au NBPs were the main generating unit of color output which greatly affected mixed color signals generated by silver deposition and growth. Additionally, their color change followed their aspect ratio and was highly dependent on the LSPR peak. Therefore, four sets of Au NBPs of different wavelengths were selected for this investigation. The morphology of monodisperse Au NBPs with four wavelengths was characterized by changing the amount of seed added to the synthesis of Au NBPs (**Figure** **3**a–d). By comparing the results within the same target concentration range, the most appropriate Au NBPs was selected. As a result of the relatively low displacement change and no significant color change, 705 and 775 nm Au NBPs cannot meet the requirements of the required sensors (Figure 3e; Figure S3a–d, Supporting Information). At 740 nm Au NBPs, the color difference value calculated from the color model of the solution color (Figure 3f) displayed the highest difference value (Figure 3g). According to these results, Au NBPs with wavelengths of 740 nm were most suitable for colorimetric sensors. ALP detection also showed an overall redshift of the UV–vis peak with color change (Figure 3h). A rod‐like morphology gradually developed during silver deposition and growth on Au NBPs also as shown in Figure S3i–l (Supporting Information). The large blue shift can reach 650 nm, which allows a wide range of quantitative analyses to be accomplished. Rd color depth impacted the following color combinations, so GSNO should be optimized. Optimization experiments were conducted on GSNO in a concentration range of 0–30 mM. After responding, the absorbance at 550 nm of Rd tended to be stable at 20 mM of GSNO as the concentration of GSNO gradually increased (Figure 3i). The solution also gradually reached saturation chroma at 20 and 30 mM GSNO concentrations (Figure 3j). Thus, 20 mM GSNO was selected as the optimal reaction condition.

### 2.3 Performance Analysis of Distinguishable Multi‐Color ALP Detection

Under optimal experimental conditions, the superiority of the developed visualization technique and ALP detection performance was analyzed. This was expected to be suitable for PCa‐BM early diagnosis. Compared with the serum ALP concentration of normal subjects (40–150 U L^−1^), the serum ALP concentration of PCa‐BM patients showed abnormally elevated expression (>157.5 U L^−1^, more than 5% above the threshold).^[^ ^11^, ^29^ ^]^ To make the distinguishable colorimetric sensor designed in this paper more suitable for rapid PCa‐BM diagnosis by the naked‐eye, 150 U L^−1^ was the threshold value. We expect that the color of this design method below and above 150 U L^−1^ can be distinguished by the naked‐eye to determine whether the ALP concentration is abnormal.

**Figure 4:**
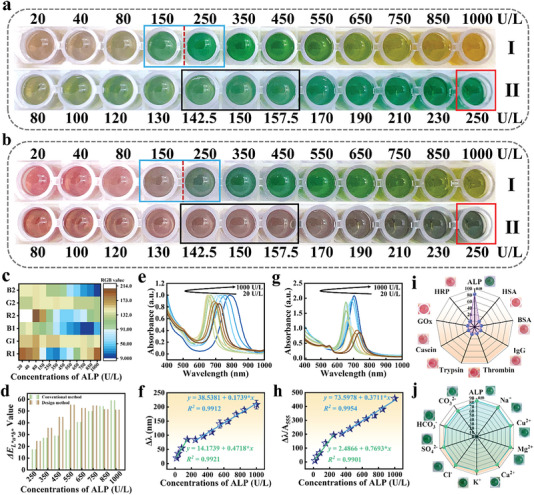
Standard colorimetric card (I: 20–1000 U L^−1^, II: 80–250 U L^−1^) for ALP detection by a) conventional colorimetry and b) DC. c) The change in RGB (R1, G1, B1) of ALP detected by conventional colorimetry is compared with the change in RGB (R2, G2, B2) of ALP detected by DC and d) the change in color difference value. The e) UV–vis absorption spectra and the corresponding f) standard curves of ALP were determined by conventional colorimetry in the concentration range of 20–1000 U L^−1^. Linear equations: *y* = 14.1739 + 0.4718*x (*R^2^* = 0.9921, 20–150 U L^−1^) and *y* = 38.5381 + 0.1739*x (*R^2^* = 0.9912, 250–1000 U L^−1^). The g) UV–vis absorption spectra and the corresponding h) standard curves of ALP were determined by DC in the concentration range of 20–1000 U L^−1^. Linear equations: *y* = 2.4866 + 0.7693*x (*R^2^* = 0.9901, 20–250 U L^−1^) and *y* = 73.5978 + 0.3711*x (*R^2^* = 0.9954, 350–1000 U L^−1^). i) Selectivity and j) anti‐interference of DC for ALP detection (inset: color photographs).

A comparison experiment was carried out with traditional colorimetric methods to verify the above design. As shown in **Figure** **4**a‐I and Figure 4b‐I, there were five color changes (rufous, brown, green, yellow‐green, yellow) and six color changes (red, red‐brown, tawny, dark green, bottle green, yellow‐green) in the process of ALP detection (20–1000 U L^−1^), respectively. According to Figure 4a‐I and b‐I, DC clearly distinguished the critical concentration at 150 U L^−1^ by an open hole, versus conventional colorimetry. This proves that these colors are a simple and intuitive means of identifying different ALP concentrations. A comparison of color results showed that dye molecules increased color variety and improved visual recognition. In order to illustrate the color change more accurately at the 150 U L^−1^ threshold, colors with ±5% concentration deviation were included in the black wireframe, which showed almost no difference in color (as illustrated in Figure 4a‐II,b‐II; Figure S5a,b, Supporting Information). Our colorimetric method can effectively distinguish red‐brown and dark green at 150 U L^−1^, whereas traditional colorimetric methods cannot distinguish green well at 150 U L^−1^. Further evidence suggests that the ALP‐induced double reaction can be used to detect abnormal ALP expression through the color enhancement effect.

This highlights the advantages of colorimetric methods for improved differentiation. A more accurate color comparison was implemented to further verify the above views. Hence, RGB and L*a*b* color models were extracted from ALP detection colors by two colorimetric methods (Figure 4c; Figure S6, Supporting Information). In the following step, the color difference value (*ΔE*) was obtained by calculating the formula: *ΔE* = [(ΔL*)^2^ + (Δa*)^2^ + (Δb*)^2^]^1/2^.^[^ ^23c^ ^]^ It was found that the color difference value of DC (*ΔΕ*: 24–55) was higher than that of conventional colorimetry (*ΔΕ*: 17–50) in ALP detection (Figure 4d). As expected, DC detected critical concentrations much better than traditional colorimetry.

For a more thorough and thoughtful analysis of detection and diagnosis requirements. As shown in Figure 4e,g, ALP was detected using conventional colorimetry and DC in a wide concentration range (20–1000 U L^−1^). Au NBPs had a blue‐shifted and red‐shifted absorption peak (722 nm‐650 nm‐787 nm; 737 nm‐653 nm‐704 nm) at different ALP concentrations, respectively. The Δλ_max_ reflected the degree of change of Au NBPs, which showed an excellent linear relationship with ALP in the concentration range of 20–150 and 250–1000 U L^−1^, 20–250 and 350–1000 U L^−1^ (Figure 4f,h), respectively. However, the difference was that our designed method had a distinct absorption peak at 550 nm for the reaction between NO and Rd (Figure 4g), and its absorbance exhibited a negative correlation with ALP concentration (Figure S4, Supporting Information). The novel colorimetric method can also generate ratio signals from two response signals and produce more accurate results when analyzing ALP concentration quantitatively.

### 2.4 Selectivity and Anti‐Interference of Distinguishable Colorimetric Sensor

In order to further explore the potential of DC, we evaluated its selectivity and anti‐interference in real samples. Many proteins similar to ALP exist in human serum, so we chose different kinds of enzymes and biological proteins (50 µg L^−1^ of ALP, HAS, BSA, IgG, HRP, GOx, thrombin, trypsin, and casein) for selective colorimetric testing. As shown in Figure 4i, the colorimetric method produced the strongest signal when detecting ALP. In contrast, the signal generated when detecting other substances is very weak and almost negligible. In addition, a variety of common anions and cations (25 mM of Na^+^, Cu^2+^, Mg^2+^, Ca^2+^, K^+^, Cl^−^, SO_4_^2−^, HCO_3_^2−^ and CO_3_^2−^) were selected for interference tests of colorimetric systems. Figure 4j shows that the interference of common anions and cations on ALP detection was controllable and reliable. Distinctive colorimetry led to excellent ALP detection performance with superior selectivity and anti‐interference.

### 2.5 Detection and Analysis of ALP Content in Osteoblasts

**Figure 5:**
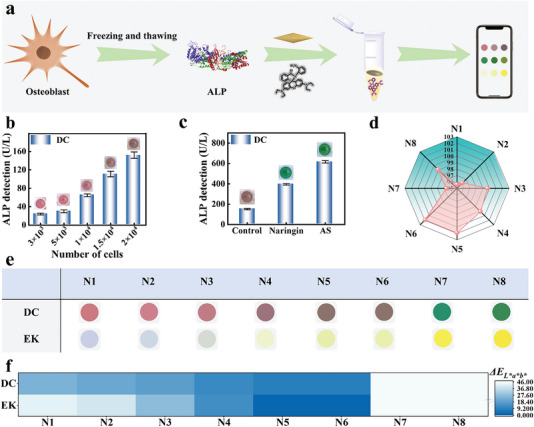
a) Schematic diagram of ALP content determination in osteoblasts. b) Changes in ALP concentration in osteoblasts with different cell numbers. c) Changes in ALP concentration in osteoblasts with different proliferation degrees. d) Comparison of ALP determination by DC and EK. e) Comparison of color change and f) color difference of ALP content in osteoblasts determined by DC and EK.

This experiment demonstrated the principle of abnormal ALP expression as a biomarker of BM and provided guidance for serum follow‐up analysis. This method was used to further examine the ALP content in osteoblast cells and colorimetric analysis was performed with osteoblast lysates instead of ALP standard samples (**Figure** **5**a). To verify the reliability of the colorimetric method, an ELISA kit (EK) was used as a first step. The linear equation was obtained by standard sample determination: *y* = 0.0015 + 0.00253*x (*R^2^* = 0.9917) (Figure S7, Supporting Information). Figure 5b shows ALP concentration was positively correlated with osteoblast proliferation. Then, naringin and alendronate sodium were selected to induce osteoblasts to monitor ALP concentration changes.^[^ ^30^ ^]^ An increase in ALP concentration was observed after lesion induction as shown in Figure 5c. The concentration varied with osteoblasts, which confirmed the abnormal increase in ALP levels after osteoblasts proliferate in BM.

More importantly, all results of the colorimetric method showed that the concentrations of ALP detected were comparable to those detected by EK (Figure S8, Supporting Information), and the concentration deviation was kept within ±5% (Figure 5d). The results show that colorimetry can identify real tumor cell samples. It is worth mentioning that the RGB and L*a*b* color models of the colors of the two methods were extracted to calculate the color difference value (DC *ΔE*: 9.1–45.9; EK *ΔE*: 0–45.1) (Figure 5f; Figure S9, Supporting Information), which clarified the reliability of the colorimetric method from the perspective of visual distinction. Clearly, these results demonstrated that the maximum color difference change obtained using this method was reliable for discrimination (Figure 5e), and it also confirmed that the color recognition method can be applied to actual samples.

### 2.6 Serum Compatibility of Distinguishable Colorimetric Sensor

The reliability of this colorimetric assay for real sample detection was further validated by adding ALP at different concentrations to PBS and serum samples. Table S1 (Supporting Information) shows spiked recovery of 95.8%−101.3% in PBS buffer and 105.5%–107.3% in serum, respectively. A number of recoveries fall within the range of reliable detection, which indicated the potential application of this colorimetric assay to real samples.

### 2.7 Analysis of Real Serum Samples and Diagnosis of PCa‐BM

**Figure 6:**
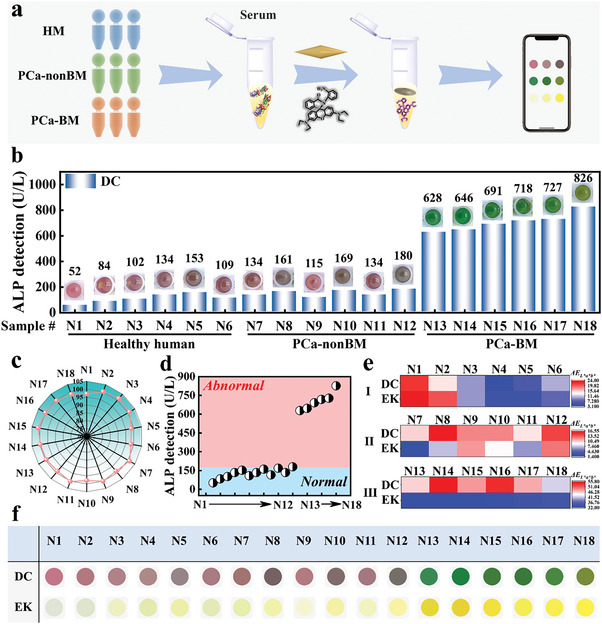
a) Schematic diagram of ALP content in serum samples of healthy volunteers and patients. b) Serum ALP content and color changes in healthy volunteers (N1–N6), PCa‐nonBM patients (N7–N12) and PCa‐BM patients (N13–N18) were measured by DC (inset: color photographs). c) The accuracy of ALP content obtained by this DC compared to EK. d) PCa‐BM screening based on ALP determination by DC. e) Samples with normal and abnormal ALP concentrations were screened by DC. f) The color difference between three serum samples (I: HM, II: PCa‐nonBM, III: PCa‐BM) determined by DC.

As part of our evaluation of the potential use of ALP colorimetric assay in clinical testing, we further quantified ALP in clinical serum samples collected from 12 cancer patients (6 patients with PCa‐nonBM and 6 patients with PCa‐BM) and 6 healthy volunteers. As shown in **Figure** **6**a, the collected whole blood samples were pretreated and centrifuged to obtain serum for colorimetric analysis and EK testing. Figure 6b shows the results of ALP in serum samples by this colorimetric method. That the ALP concentration was quantitatively determined by analyzing Au NBPs LSPR peak shift. According to the results detected by the designed method, the accuracy was between 97% and 101.4%, which indicated that the method would be able to detect similar amounts and be as accurate as the EK (Figure 6c; Figure S10, Supporting Information). It is clear that this colorimetric method has the potential for reliable and accurate practical applications.

The above studies also showed that this colorimetric method could effectively screen for BM in real serum samples (Figure 6d). Where PCa‐BM samples had higher ALP concentrations than PCa‐nonBM and healthy samples. RGB and L*a*b* color patterns (Figure S11, Supporting Information) were extracted for color difference calculation, and it was found that the color difference value of this colorimetric method (*ΔE*: I 3.2–21.3; II 9.9–16.5; III 41.6–55.8) was significantly higher than that of the kit (*ΔE*: I 4.7–24; II 1.4–12.7; III 32.1–35.1) in serum detection of patients (Figure 6e), indicating that our method was more convenient for simple and rapid diagnosis of disease with naked eyes. In order to further demonstrate the superiority of this colorimetric method with the naked eye, the colors of the detection results of the two methods were further compared in Figure 6f. In contrast to the monochromatic EK variation, the sensor can display different colors for evaluation of different types of clinically practical samples. Moreover, PCa‐BM response results were significantly different from PCa‐nonBM and healthy samples. This also suggested that a preliminary diagnosis of a sample can be made directly from visual observation. Consequently, we believe that the ALP colorimetric sensor developed through distinguishable colorimetric methods can be useful for portable PCa‐BM diagnosis.

## 3 Conclusion

Based on distinguishable colorimetric analysis, we developed an ultrasensitive naked‐eye detection for PCa‐BM patients with abnormal ALP concentrations. In comparison to silver deposition on Au NBPs, the prepared Rd composite probe provided more abundant visual information. As a result, naked‐eye detection has been dramatically transformed from a qualitative/semi‐quantitative mode to a quantitative mode with excellent accuracy. A critical concentration of 150 U L^−1^ can clearly be distinguished. It was demonstrated that this novel colorimetric analysis method can be used for clinical diagnostic purposes. ALP concentrations in PCa‐BM patients can also be detected and diagnosed by observing distinct color changes with the naked eye. By means of this study, we provided a broad avenue for rationally designing and developing the next generation of high‐performance visual detection.

## 4 Experimental Section

### Materials and Reagents

Chlorauric acid trihydrate (HAuCl_4_·3H_2_O, 99.9%) was bought from Sigma–Aldrich Co., Ltd. (Shanghai). Cetyltrimethyl ammonium bromide (CTAB, 99.0%) and Cetyltrimethyl ammonium chloride (CTAC, 99.0%) were purchased from TCI. Trisodium citrate (SC, ≥ 99.0%), sodium borohydride (NaBH_4_, 99.0%), silver nitrate (AgNO_3_, > 99.0%), hydrochloric acid (HCl, 37 wt.%), aqueous ammonia (NH_3_·H_2_O, 30 wt.%), hydrogen peroxide (H_2_O_2_, 30 wt.%), dichloromethane (DCM, 99.0%) were purchased from Sinopharm Chemical Reagent Co., Ltd. (Shanghai, China). Sodium orthovanadate (Na_3_VO_4_, 99.7%), AA (99.7%), glucose oxidase (GOx, 100 U mg^−1^) were purchased from Macklin Biochemical Co., Ltd. (Shanghai, China). AAP (99.0%) were purchased from Zesheng Technology Co., Ltd. (Anhui, China). ALP (≥ 10 units mg^−1^), ALP buffer (2X, pH 7.5, R20997), recombinant human serum albumin (HAS, 95.0%, S29747), thrombin enzyme (1 kU, S10117), human immunoglobulin (IgG, S25766), casein peptone (S23107), bovine serum albumin (BSA, 98.0%, S12012), trypsin enzyme (S10033) were purchased from Yuanye Biotechnology Co. Ltd. (Shanghai, China). Horseradish peroxidase (HRP) was purchased from Sangong Biotech Co., Ltd. (Shanghai, China). S‐Nitrosylglutathione (GSNO) and Human ALP EK was purchased from Huzhen Industrial Co., Ltd (Shanghai, China). Mouse embryonic osteoblast progenitor cells (MC3T3‐E1 Subclone) were purchased from Haoke Biotechnology Co., Ltd. (Hangzhou, China). Serum samples from prostate cancer patients and healthy volunteers were collected at Ningbo First Hospital. None of the chemicals were further purified, and deionized water was used throughout the entire experiment.

### Instruments

Transmission electron microscopy (TEM) was performed on a JEOL HT‐7700 electron microscope operating at 200 kV (Hitachi, Japan). Field emission scanning electron microscope (FESEM, SUPRA55‐CARL ZEISS, Germany). UV–vis absorption spectra were recorded using a TU‐1810 UV–vis spectrophotometer provided by Purkinje General Instrument Co., Ltd (Shanghai, China). The precision acidity meter was provided by Dapu Instruments Co., Ltd. (Shanghai, China). The milliq ultrapure water system was offered by Millipore (Bedford, USA). The analytical balance was provided by Sartorius Scientific Instrument Co., Ltd. (Beijing, China).

### Synthesis of Au NBPs

The synthesis procedure of Au NBPs with slight modification according to the previously reported literature.^[^ ^31^ ^]^ 1) First, the citrate‐stabilized seed solution was prepared as follows: 125 µL of 0.01 M HAuCl_4_, 0.25 µL of 0.01 M trisodium citrate, 9.625 mL of deionized water and 600 µL of 0.01 M freshly prepared ice‐cold NaBH_4_ were added to a 20 mL glass bottle in turn under vigorous stirring at 1200 rpm for 2 min. Lastly, the resultant seed solution was kept at 30 °C for 2 h before use. 2) The typical procedure of Au NBPs preparation: initially, the CTAB growth solution was prepared by sequential addition of 4 mL of 0.01 M AgNO_3_, 20 mL of 0.01 M HAuCl_4_, 3.2 mL of 0.1 M AA and 8 mL of 1 M HCl into 0.4 L of 0.1 M aqueous CTAB solution under a water bath at 30 °C and stirring at 700 rpm. 5 mL of the seed solution was then added into the growth solution, followed by gentle inversion mixing for 30 s. The reaction solution was left undisturbed over 6 h at 30 °C, the unpurified Au NBPs was obtained.

### Purification of Au NBPs

The unpurified Au NBPs sample was divided into 12 tubes (50 mL) on average and centrifuged three times at 8000 rpm for 10 min. Subsequently, the precipitate of each tube was redispersed in 30 mL of 0.1 M CTAC solution. This was followed by the addition and mixing of 6 mL of 7.5 mM AgNO_3_ and 3 mL of 0.1 M AA. The resultant solution of 12 tubes was kept in an oven at 65 °C for 4 h, during which Ag was overgrown on the Au nanocrystals to produce bimetallic Au@Ag products. The spherical (Au core)@(Ag shell) nanoparticles remained in the supernatant, while the Au@Ag heteronanorods agglomerated together and precipitated to the bottom of the container. The remaining Au@Ag heteronanorods were redispersed in 20 mL water after removing the supernatant. The resultant solution was mixed gently with 1.2 mL of 30 wt.% NH_3_·H_2_O and 0.8 mL of 1 M H_2_O_2_ and kept undisturbed for 0.5 h. During this process, (Au core)@(Ag shell) nanoparticles gradually transformed into separate Au NBPs after the Ag segments were gradually etched away. Finally, the resultant solution was spun down two times at 9000 rpm for 10 min to obtain pure Au NBPs (the precipitate of each tube was dispersed in 2 mL of water).

### Synthesis of NO Recognition Molecules, Rd

The synthesis procedure of Rd according to the previously reported literature.^[^ ^32^ ^]^ L‐phenylenediamine (0.86 g) and rhodamine B (1 g) were dissolved in DCM (30 mL). Triethylamine and (1.5 mL) and 1‐propylphosphate cyclic anhydride DMF solution (3 mL, 50 wt.%) were added into the above mixture, and subsequently reacted at 50 °C for 12 h. The crude product was purified by silica gel chromatography (CH_2_Cl_2_: MeOH = 30:1, v/v) to obtain the NO recognition molecules Rd.

### Detection of ALP in PBS

Specifically, 107 µL of 10 mM CTAC, 10 µL of AAP (10 mM), 10 µL of ALP (20, 40, 80, 150, 250, 350, 450, 550, 650, 750, 850, and 1000 U L^−1^), 50 µL of AgNO_3_ (10 mM) and 50 µL of Au NBPs were added to the 0.5 mL of tube in turn and then kept at 37 °C for 10 min. Whereafter, 3 µL of GSNO (20 mM) and 50 µL of Rd (1 mg mL^−1^) were added to the above solution and kept at 37 °C for 5 min. The test solution was analyzed to determine the variation in UV–vis spectrum and color after 5 µL Na_3_VO_4_ (0.1 M).

### Osteoblast Proliferation

The second‐generation osteoblasts were inoculated into the culture dish at a concentration of 2 × 10^4^ cells mL^−1^. When the cells were 80% fused, the original medium was abandoned and the serum‐free DMEM medium containing alendronate sodium (1.0 × 10^−8^ mol L^−1^)/naringin (100 µM) was added, and the cultured cells were placed in the incubator (5% CO_2_, 37 °C) for 48 h.

### Detection of ALP in Osteoblasts

After osteoblast proliferation culture, the medium was abandoned and cleaned with PBS three times. The cells were repeatedly freeze‐thawed three times for cell lysis and then the cell lysis fluid was collected for further use. Following that, ALP of cell lysis fluid was detected by EK and DC.

### Test of Full Blood Sample

In order to preserve the supernatant for future use, whole blood samples were centrifuged at 3000 rpm for 5 min. The above procedure was repeated three times, serum samples were obtained. Lastly, the ALP in the serum samples was tested by EK and DC. This study was approved by Ningbo First Hospital's Institutional Ethical Committee (No. 013A‐01, 2022). The experiments were carried out with the full, informed consent of the subjects.

### Color Analysis

The CIELAB color space was defined by the International Commission on Illumination (CIE) in 1976, and this color model was commonly used to describe all colors visible to the human eyes. In CIELAB, the color is represented by three parameters which include brightness (L*) and color channels (a* and b*). The perceptual distance (color difference value) *ΔE* = [(ΔL*)^2^ + (Δa*)^2^ + (Δb*)^2^]^1/2^ provides a measure of how far apart and how much contrast, exists between two colors. CIELAB was intended as a perceptually uniform space in which a given number change corresponds to a perceived color change. The color photographs can be obtained by a smartphone. Then RGB, L*, a* and b* values of the color photos were extracted by the software (Adobe Photoshop CS6). Changes in three parameters intensity (ΔL*, Δa*, Δb*) of samples were collected before and after detection at each controlled condition. Finally, *ΔE* was calculated according to the above Eq.

## Conflict of Interest

The authors declare no conflict of interest.

## Supporting information

Supporting Information

## Data Availability

Research data are not shared.
