# Supplementary material for: Distinguishable Colorimetric Biosensor for Diagnosis of Prostate Cancer Bone Metastases
Source: Adv Sci (Weinh). 2023 Oct 15;10(32):2303159. doi: 10.1002/advs.202303159 (PMC10646272; doi:10.1002/advs.202303159)
Supplement: Supplementary file 1 — Supporting Information [file ADVS-10-2303159-s001.pdf]

ADVANCED SCIENCE
Open Access

Supporting Information

for *Adv. Sci.*, DOI 10.1002/advs.202303159

Distinguishable Colorimetric Biosensor for Diagnosis of Prostate Cancer Bone Metastases

Ming Li, Caiping Ding, Dong Zhang, Weiwei Chen, Zejun Yan, Zikang Chen, Zhiyong Guo*, Longhua Guo and Youju Huang**

Supporting Information

Distinguishable Colorimetric Biosensor for Diagnosis of Prostate Cancer Bone Metastases

Ming Li,[a,b#] Caiping Ding[b#] Dong Zhang,[a] Weiwei Chen,[b] Zejun Yan[a],Zikang Chen,[b] Zhiyong Guo,[c*] Longhua Guo,[d] Youju Huang[b*]*

[a] Department of Urology & Nephrology, The First Affiliated Hospital of Ningbo University, 59, Liuting Street, Ningbo, 315010, Zhejiang, China

[b] Department Hangzhou Normal University, College of Material Chemistry and Chemical Engineering, Key Laboratory of Organosilicon Chemistry and Material Technology, Ministry of Education, Key Laboratory of Organosilicon Material Technology of Zhejiang Province, Hangzhou 311121, Zhejiang, China

[c] State Key Laboratory for Managing Biotic and Chemical Threats to the Quality and Safety of Agro-products, State Key Laboratory Base of Novel Functional Materials and Preparation Science, School of Materials Science and Chemical Engineering, Ningbo University, Ningbo, 315211, Zhejiang, China

[d] College of Biological, Chemical Sciences and Engineering, Jiaxing University, Jiaxing, 314001, Zhejiang, China

# Ming Li and Caiping Ding contributed equally to this work.

*Corresponding author:
Zejun Yan, E-mail: fyyyanzejun@nbu.edu.cn
Zhiyong Guo, E-mail: guozhiyong@nbu.edu.cn
Youju Huang, E-mail: yjhuang@hznu.edu.cn

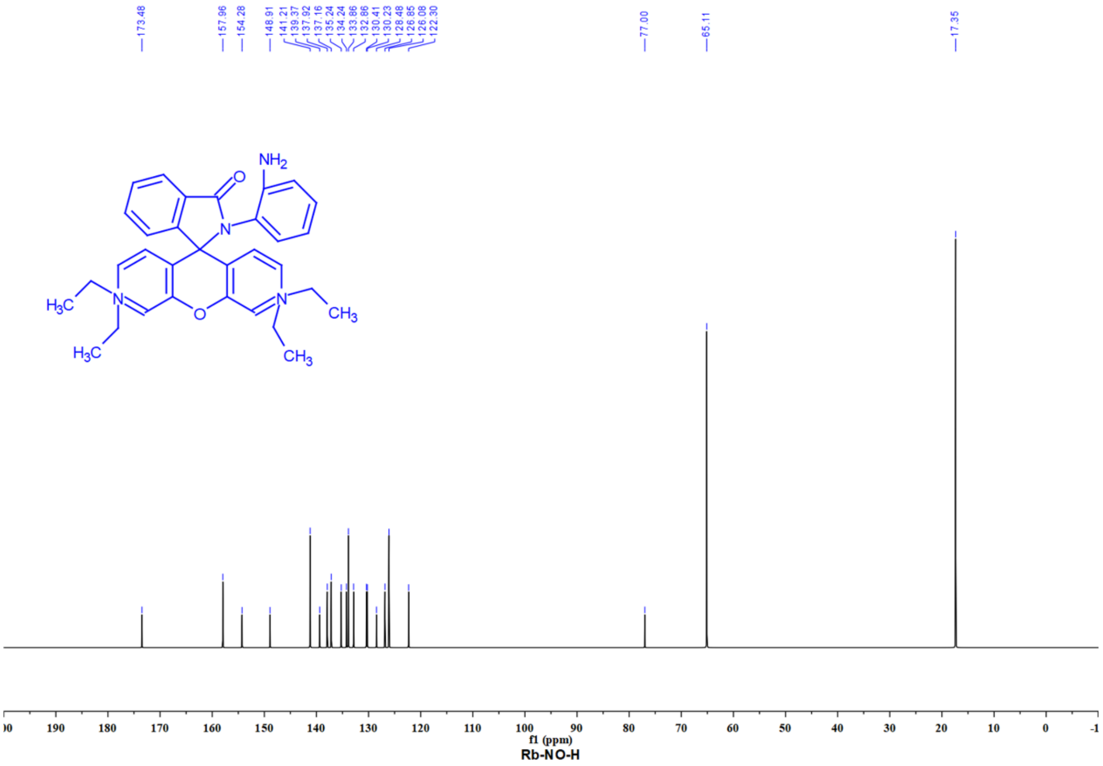

Figure S1. ^{1}H-NMR spectrum of Rd molecules.

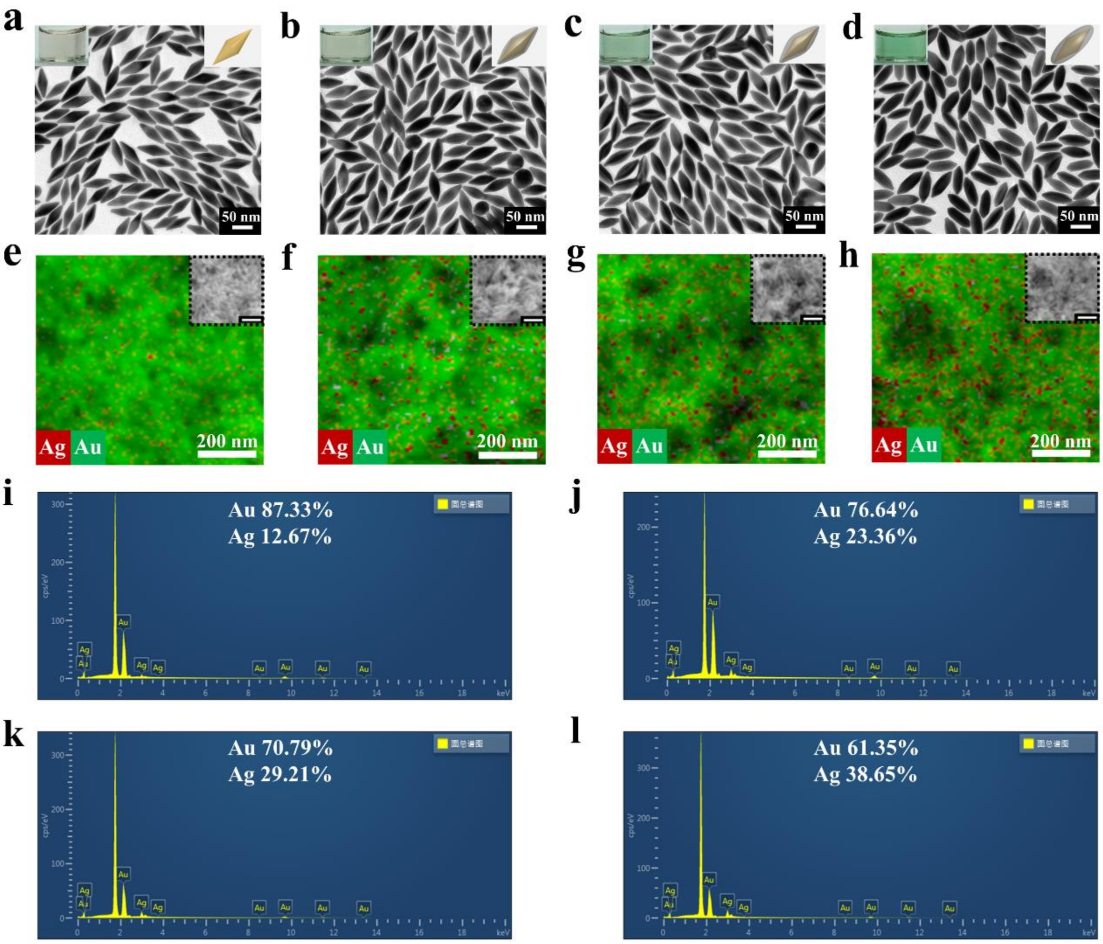

Figure S2. (a-d) TEM images and corresponding (insets: solution color and morphological model of Au NBPs) (e-l) elemental analysis diagram (insets: SEM images of Au NBPs after growth) of Au NBPs before and after growing under different concentrations of ALP: 0, 100, 200 and 300 U/L.

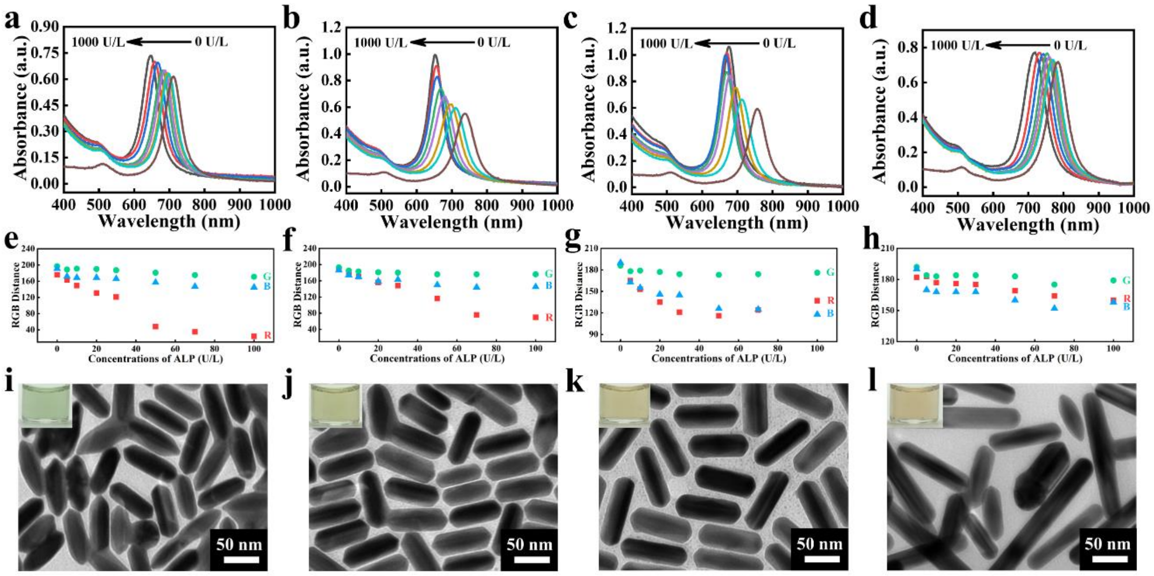

Figure S3. In the presence of 0-1000 U/L ALP, (a-d) UV-vis spectra, (e-h) changes of RGB and (i-l) TEM images of Au NBPs at four wavelengths (705 nm, 740 nm, 756 nm and 775 nm) after silver deposition and growth on Au NBPs.

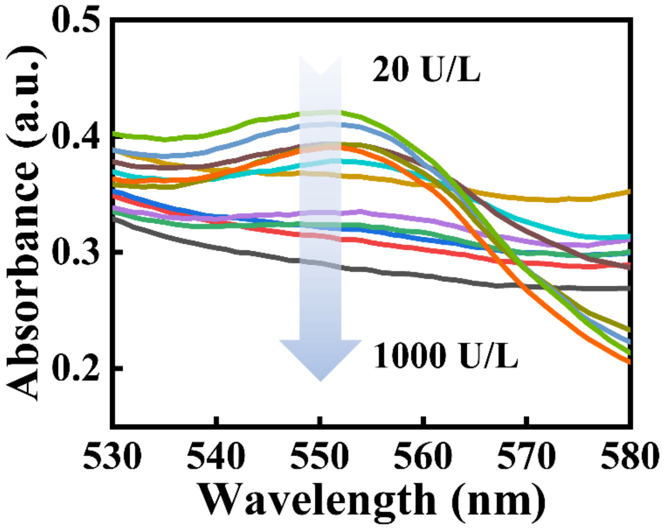

Figure S4. The UV-vis spectra of Rd during ALP concentrations were detected by hybrid colorimetry in range of 20-1000 U/L.

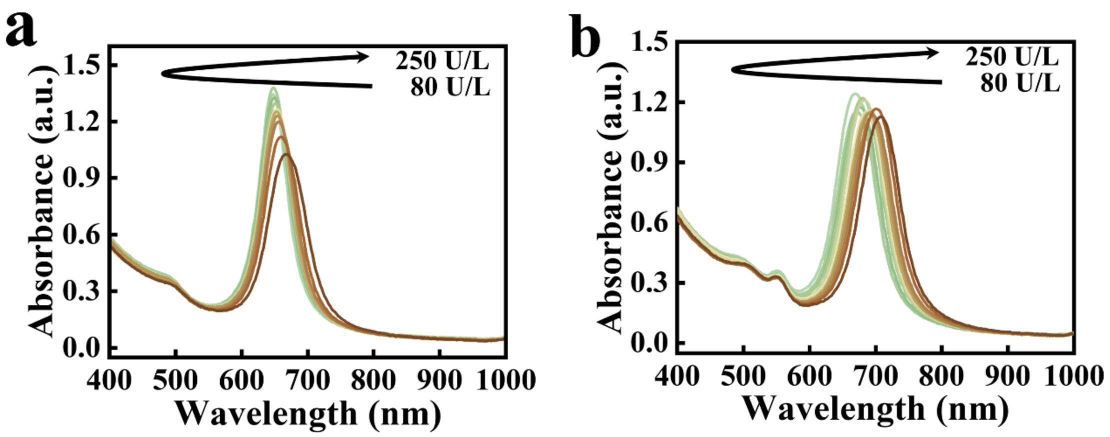

Figure S5. The UV-vis spectra of Au NBPs in the process of ALP detected by (a) traditional colorimetry and (b) DC in the range of 80-250 U/L.

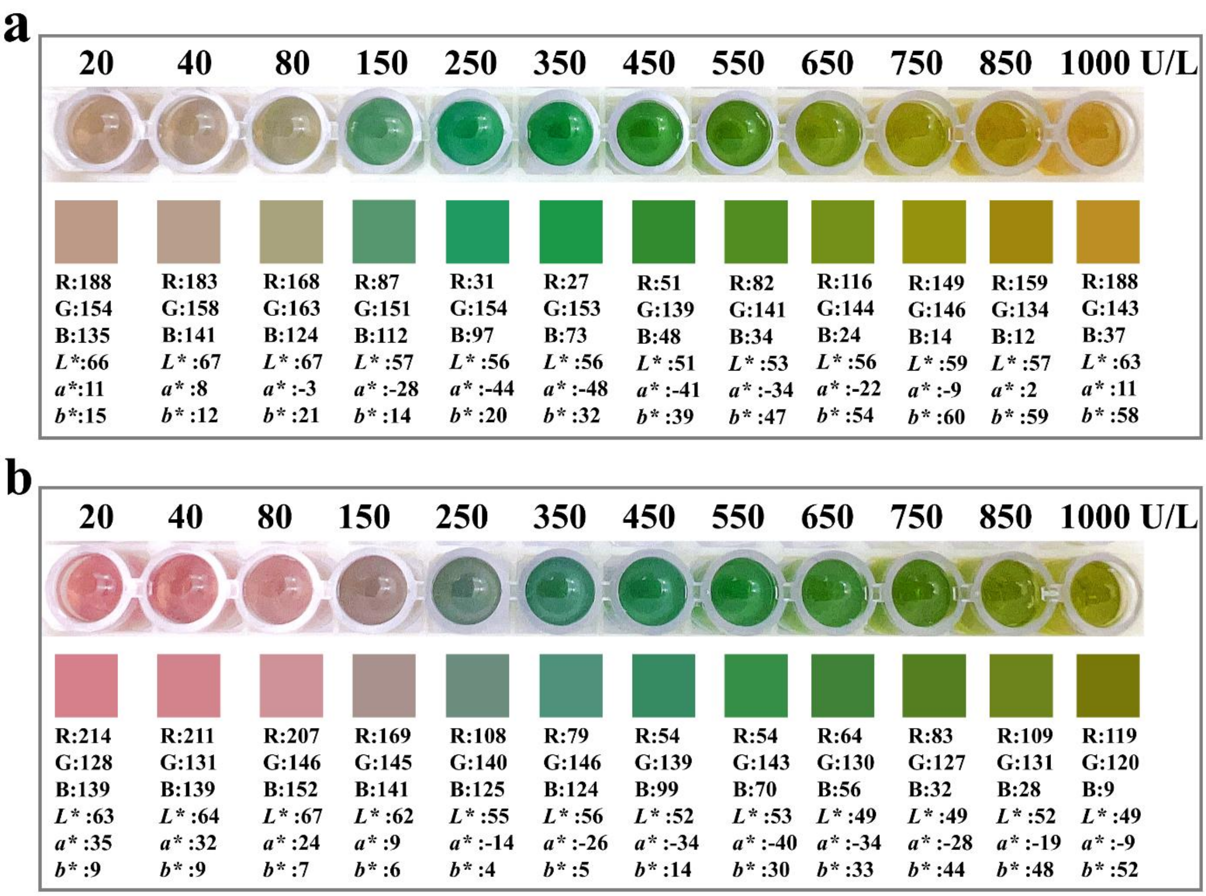

Figure S6. RGB and $L^*a^*b^*$ color models be extracte from standard colorimetric cards of (a) conventional colorimetry and (b) DC (ALP detection in range of 20-1000 U/L).

Table S1. Analytical performance of this sensor in detecting ALP in real samples.

samples	color change	Δλ (nm)	Spiked (U/L)	Found (U/L)	Recovery (%)	RSD (n=3, %)
PBS 1	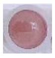	34.0	80	76.6	95.8	1.5
PBS 2	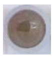	39.0	150	152.0	101.3	2.8
PBS 3	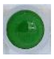	71.0	550	537.5	97.7	2.4
serum 1	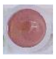	35.0	80	84.7	105.9	4.7
serum 2	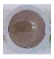	40.0	150.0	161.0	107.3	5.9
serum 3	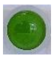	78.0	550	580.1	105.5	5.3

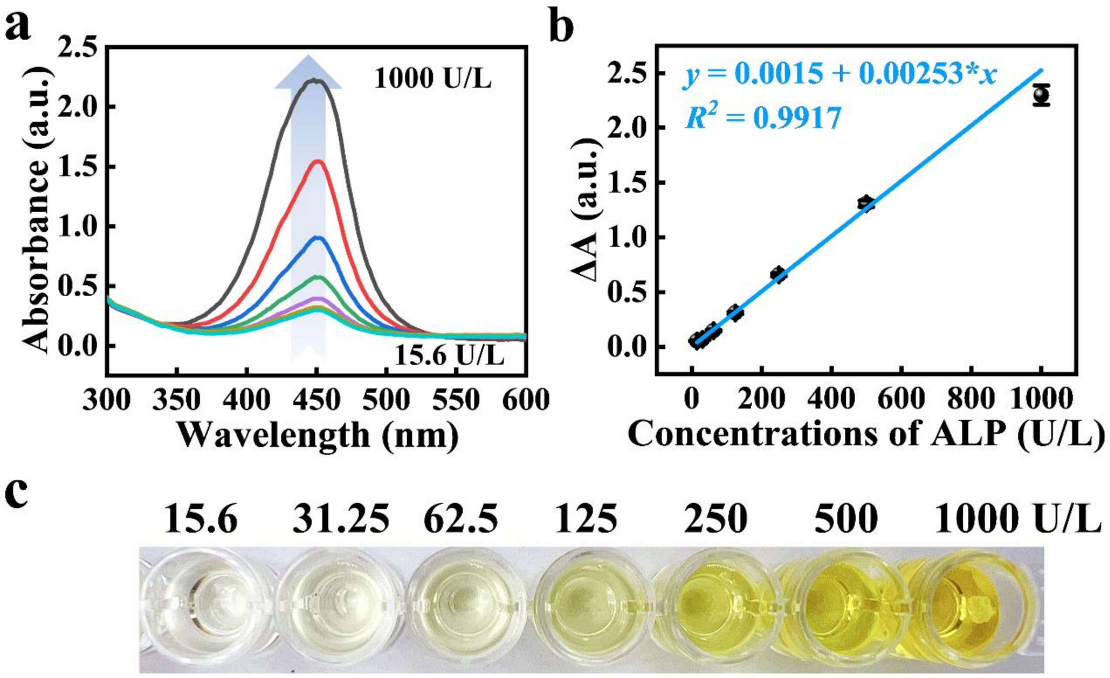

Figure S7. (a) UV-vis spectra, (b) standard curve and (c) color changes obtained by the determination of standard samples of ALP EK.

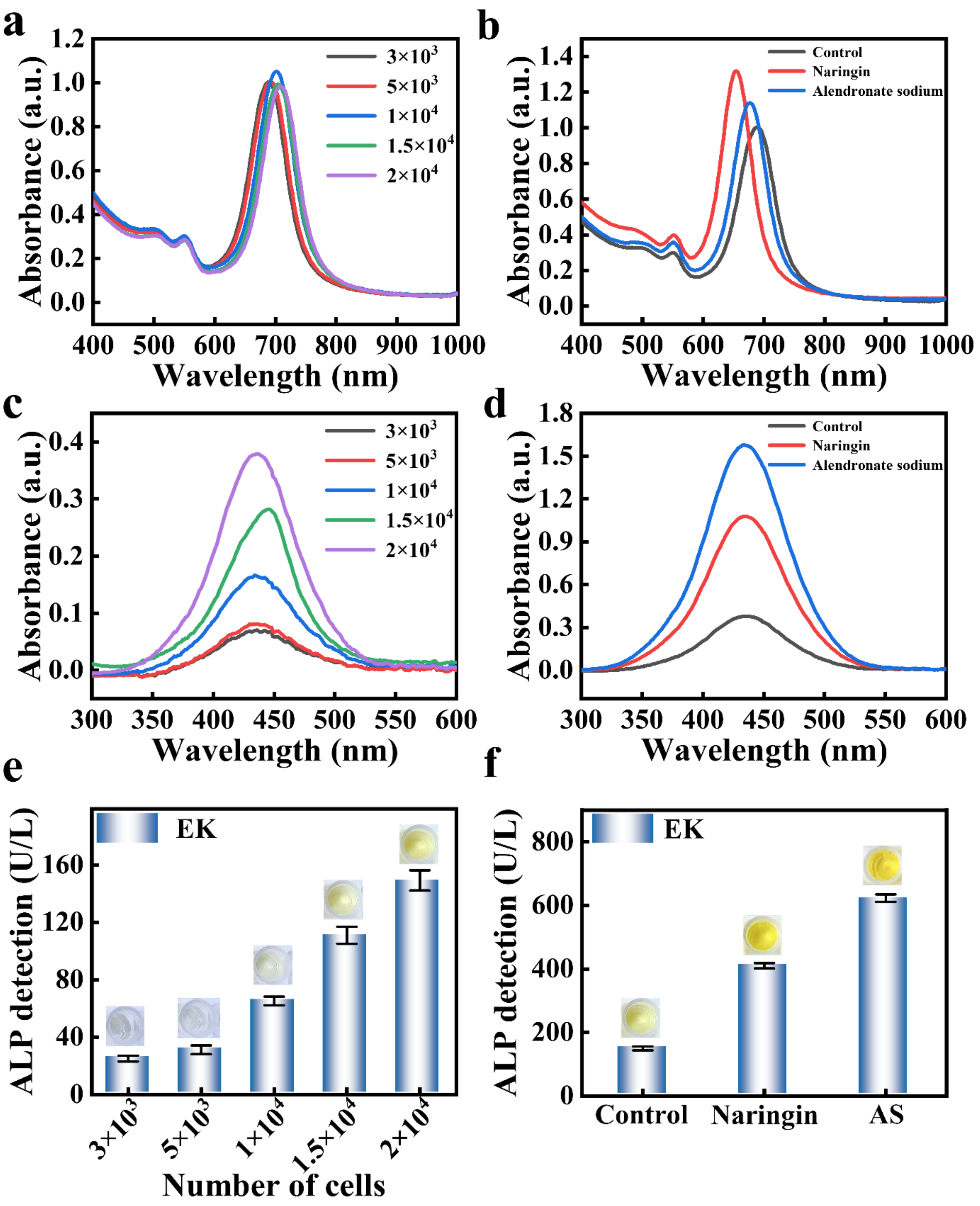

Figure S8. UV-vis spectra of ALP concentrations detection of osteoblasts with (a) different cell numbers and (b) different degrees of proliferation by DC. UV-vis spectra of ALP concentrations detection of osteoblasts with (c) different cell numbers and (d) different degrees of proliferation by EK. Columnar statistical chart of ALP concentrations detection of osteoblasts with (e) different cell numbers and (f) different degrees of proliferation by EK.

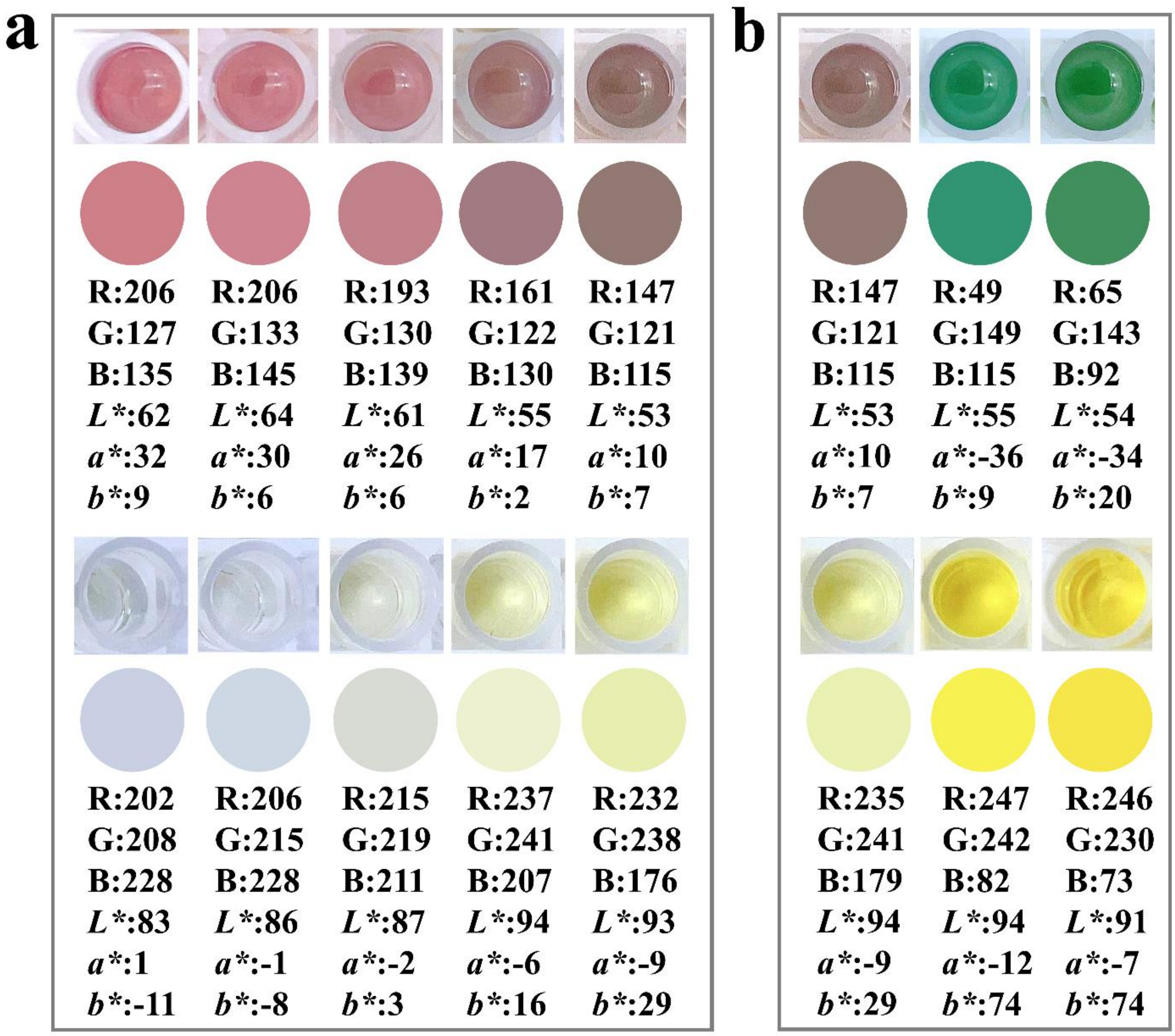

Figure S9. RGB and *L*a*b** models were extracted from the color in the process of ALP concentrations detection of osteoblasts with (a) different cell numbers and (b) different degrees of proliferation by DC and conventional colorimetry.

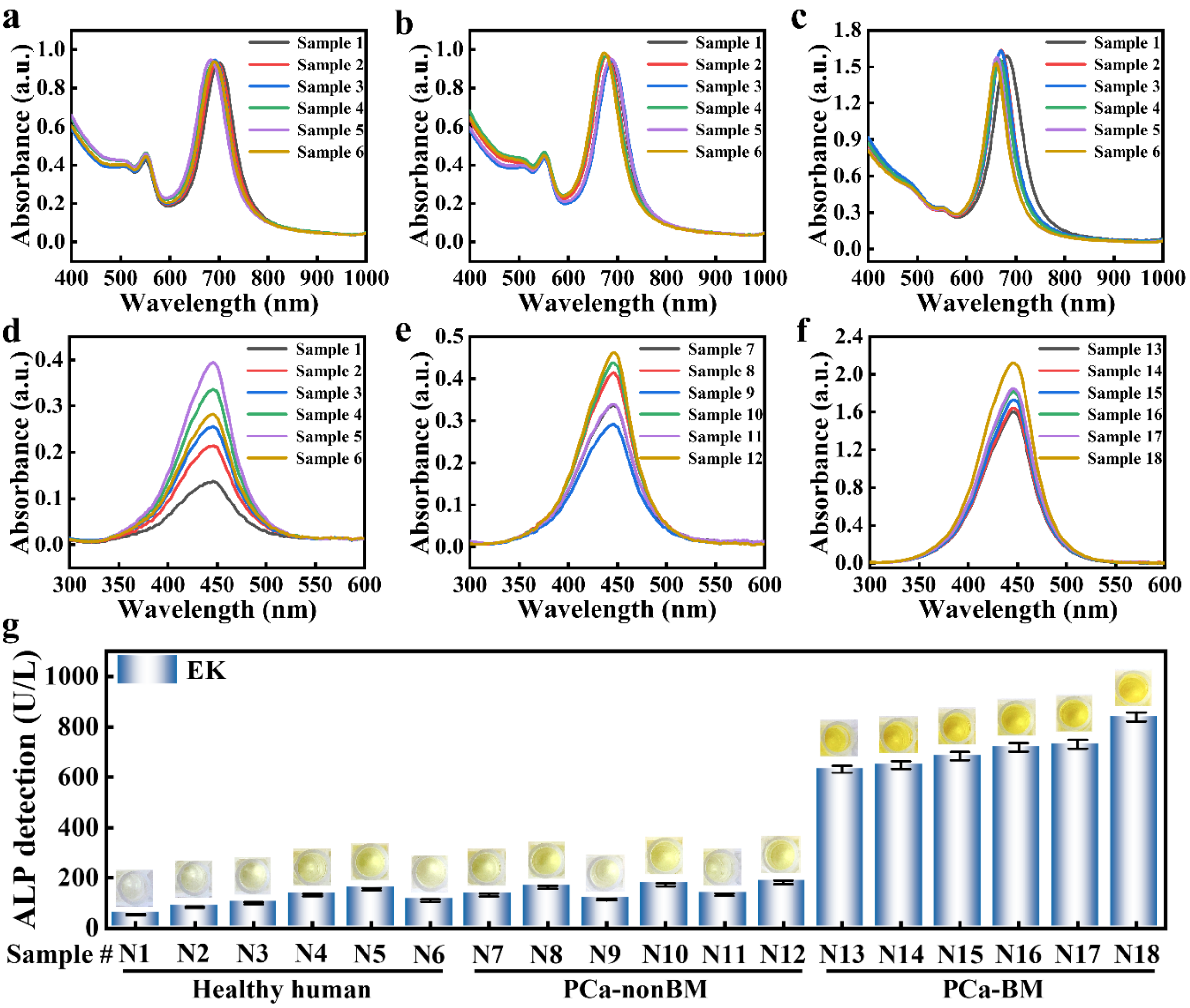

Figure S10. UV-vis spectra of ALP concentrations detection of HM (a), PCa-nonBM (b) and PCa-BM (c) serum samples by DC. UV-vis spectra of ALP concentrations detection of HM (d), PCa-nonBM (e) and PCa-BM (f) serum samples by EK. (g) Columnar statistical chart of the detection result of EK.

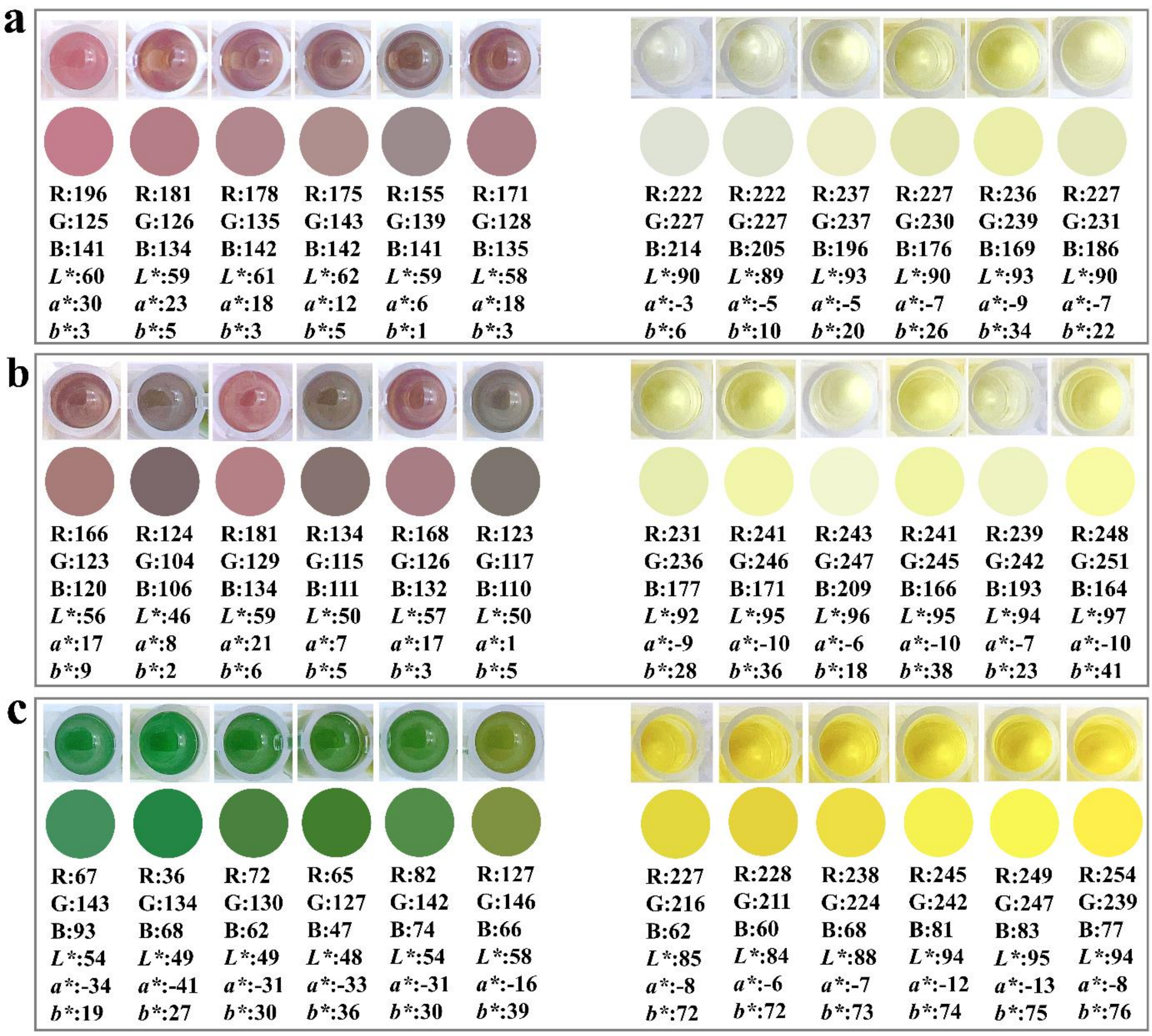

Figure S11. RGB and $L^*a^*b^*$ models were extracted from the color in the process of ALP concentrations detection of (a) HM, (b) PCa-nonBM and (c) PCa-BM serum samples by DC and conventional colorimetry.
